# Age-Dependent Outcomes of Reductive Ascending Aortoplasty

**DOI:** 10.3390/medicina62040672

**Published:** 2026-04-01

**Authors:** Živojin S. Jonjev, Ilija Bjeljac, Anđela Božić, Mirko Todić, Kristina Jonjev, Aleksandar M. Milosavljević, Jovan Rajić, Strahinja Mrvić

**Affiliations:** 1 Institute for Cardiovascular Diseases of Vojvodina, Clinic of Cardiovascular Surgery, 21204 Sremska Kamenica, Serbia; ilija.bjeljac@ikvbv.ns.ac.rs (I.B.); andjela.bozic.324b21@gmail.com (A.B.); mirko.todic@mf.uns.ac.rs (M.T.); amk88i@gmail.com (A.M.M.); jovan.rajic@ikvbv.ns.ac.rs (J.R.); strahinja.mrvic@ikvbv.ns.ac.rs (S.M.); 2 Faculty of Medicine, University of Banja Luka, 78000 Banja Luka, Bosnia and Herzegovina; 3 Faculty of Medicine, University of Novi Sad, 21102 Novi Sad, Serbia; kristinajonjev@gmail.com

**Keywords:** ascending aorta, aortic aneurysm surgery, reductive aortoplasty, aortic valve disease

## Abstract

*Background and Objectives*: The optimal management of dilated ascending aorta during aortic valve replacement (AVR) in older or high-risk patients remains debated. While graft replacement is the standard procedure, reductive ascending aortoplasty (RAA) may offer a less invasive, tissue-preserving alternative. This study evaluated long-term RAA outcomes and identified the optimal patient profile. *Materials and Methods*: In a single-center cohort, 64 patients underwent AVR with unwrapped RAA (2005–2025). Patients were stratified by valve phenotype (tricuspid [TAV], n = 45; bicuspid [BAV], n = 19) and age (<70 years, n = 52; ≥70 years, n = 12). Endpoints were early safety, long-term survival, and aortic redilatation (≥50 mm). *Results*: Outcomes diverged markedly by subgroup. Patients aged ≥70 years demonstrated excellent 10-year freedom from redilatation (83.3%) with no reinterventions. In contrast, BAV patients had higher redilatation rates (31.6% vs. 8.9%; *p* = 0.053) and a trend toward more reexploration for bleeding (15.8% vs. 6.7%; *p* = 0.109). Redilatation and reintervention were concentrated in patients <70 years. *Conclusions*: RAA with AVR offers favorable long-term durability, but success is highly age-dependent. The procedure is a safe, effective tissue-preserving strategy for selected older patients (≥70 years), particularly those with TAV. However, high redilatation rates in BAV patients suggest that RAA should be avoided in this population, reinforcing graft replacement as preferred for younger patients.

## 1. Introduction

Dilated ascending aortas are a major risk factor for dissection and rupture [1,2]. Guidelines recommend graft replacement when thresholds are reached, especially in bicuspid aortic valve (BAV) patients [3,4,5]. Although prosthetic replacement is durable, it requires longer operative times and may increase perioperative risk—a consideration of paramount importance in the growing population of older and medically complex patients. This trade-off is critical for older/high-risk patients, whose frailty and limited life expectancy make prolonged clamp times less desirable and graft durability less necessary [6,7].

Current guidelines from the European Association for Cardio-Thoracic Surgery (EACTS), the Society of Thoracic Surgeons (STS), and the European Society of Cardiology (ESC) recommend prophylactic replacement of the ascending aorta when the diameter reaches 55 mm in patients with tricuspid aortic valves and 50–55 mm in patients with bicuspid aortic valves (BAVs) [3,4]. Importantly, these guidelines identify specific situations where surgery is recommended at lower thresholds (≥45 mm), including patients undergoing concomitant aortic valve replacement (AVR) with a bicuspid aortic valve or those with rapid growth (>3 mm/year) [4,5]. These thresholds are based on large population studies demonstrating that the risk of aortic dissection increases exponentially once diameters exceed 55 mm [1,2]. However, the optimal management of patients with moderately dilated aortas (45–55 mm) remains debated, especially in older or high-risk individuals for whom the risks of extended operative time may outweigh the benefits of prophylactic replacement.

A technically simpler procedure like reductive ascending aortoplasty (RAA) may therefore offer a favorable risk–benefit profile for this specific vulnerable subgroup, provided its long-term durability is acceptable. RAA avoids prosthetic material and preserves native elasticity, potentially maintaining Windkessel function [8]. The technique of RAA was first introduced by Robicsek in 1982 as a method to reduce the diameter of the ascending aorta while preserving native tissue [8]. The original description involved a longitudinal resection of an elliptical segment of the anterior aortic wall followed by linear closure. This “cut and sew” approach was subsequently modified by various authors, with some advocating external reinforcement to prevent late redilatation [9,10]. Despite initial enthusiasm, concerns regarding long-term durability emerged, particularly in younger patients and those with bicuspid aortic valves (BAVs), leading to a decline in its use in favor of prosthetic graft replacement. However, recent reports have suggested that in carefully selected patients—particularly older individuals with limited life expectancy—RAA may offer acceptable long-term outcomes with the added benefit of preserved aortic compliance [11,12].

**Figure 1:**
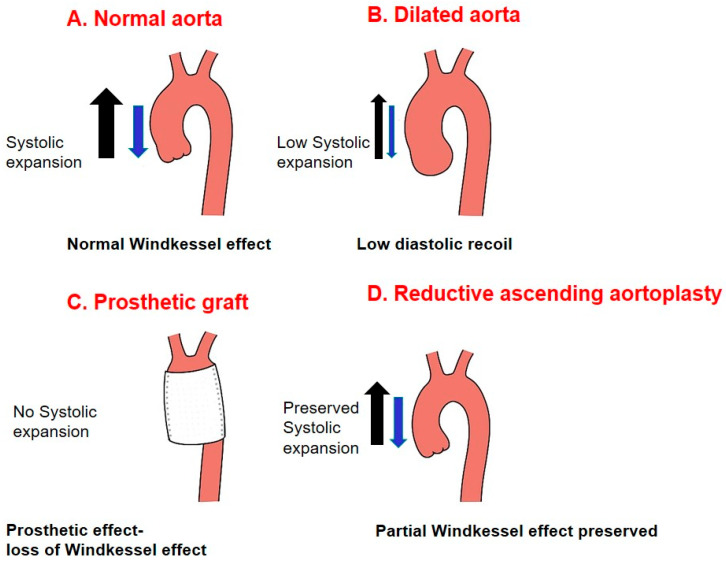
Conceptual illustration of aortic compliance and the Windkessel effect: (**A**) Normal aorta with physiological Windkessel function. (**B**) Dilated aorta with impaired compliance and buffering. (**C**) Prosthetic graft replacement, which abolishes native aortic compliance. (**D**) Reductive aortoplasty (RAA), which aims to preserve native tissue and partial Windkessel function.

A critical gap remains in understanding which patient subgroups derive durable benefit from RAA and which are at excessive risk for failure. Recent recognition of the aorta as an active organ has renewed interest in tissue preservation [13,14,15]. Through the Windkessel effect, the aorta buffers flow and perfusion; rigid grafts abolish this, while RAA may retain it (Figure 1).

The Windkessel effect, first described by Stephen Hales in the 18th century, describes the aorta’s ability to store elastic energy during systole and release it during diastole, thereby maintaining peripheral perfusion and coronary blood flow. This physiological property is progressively lost with aortic dilatation and stiffening, contributing to widened pulse pressure, increased left ventricular afterload, and ultimately myocardial dysfunction [13,14,15]. Prosthetic grafts, while structurally durable, are inherently non-compliant and therefore abolish this buffering function. In contrast, RAA preserves native aortic tissue and may partially restore or maintain the Windkessel effect—a theoretical advantage that remains largely unproven in clinical practice.

The present study reports our 20-year single-center experience with the unwrapped “cut and sew” RAA technique performed in combination with aortic valve replacement. We aimed not only to evaluate early and long-term outcomes but, more importantly, to perform a rigorous risk-stratification analysis to identify the ideal candidates for this tissue-preserving strategy and to define populations in whom it should be avoided.

## 2. Materials and Methods

### 2.1. Study Design and Patient Population

This Institutional Review Board (IRB)-approved, single-arm retrospective study evaluated patients undergoing unwrapped RAA (2005–2025) per the European Association for Cardio-Thoracic Surgery (EACTS), Society of Thoracic Surgeons (STS), and European Society of Cardiology (ESC) guidelines [3,4,5].

Excluded cases were emergencies, reoperations, and connective tissue disorders. RAA was performed exclusively as a concomitant procedure with aortic valve replacement (AVR). In a subset of high-risk patients (age ≥ 70, frailty, organ dysfunction, prior radiation) with aortic diameter ≥ 50 mm, RAA was employed as a risk-adapted alternative to more extensive graft replacement.

Patients were considered candidates for RAA if they had a moderately dilated ascending aorta (45–55 mm) with no evidence of connective tissue disease, rapid preoperative growth (>5 mm/year), or significant aortic insufficiency that would independently warrant root replacement.

### 2.2. Age Stratification

For subgroup analysis, patients were divided according to age. A cutoff of 70 years was chosen to define the “older” cohort, consistent with prior reports in cardiac and aortic surgery identifying age ≥ 70 as a threshold for increased operative risk and comorbidity burden. Patients < 70 years comprised the “younger” cohort. This stratification was prespecified as the primary subgroup analysis.

### 2.3. Valve Phenotype (Exploratory Analysis)

Patients were also classified by native aortic valve phenotype as BAV or tricuspid aortic valve (TAV), based on preoperative echocardiography and intraoperative inspection. The BAV cohort consisted of 19 patients. Given the limited sample size of this subgroup, comparative analyses involving valve phenotype were considered exploratory and are presented primarily with descriptive statistics.

### 2.4. Surgical Technique

All procedures were performed via median sternotomy. The aortic cannula was inserted into the proximal transverse arch, and cardiopulmonary bypass was instituted with mild systemic hypothermia (32–34 °C). Myocardial protection was achieved with antegrade cold (4 °C) crystalloid cardioplegia, re-administered at 20 min intervals, supplemented with topical cooling.

**Figure 2:**
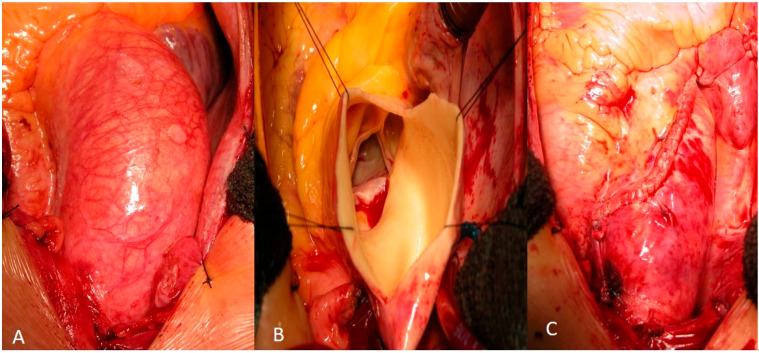
Surgical technique of unwrapped “cut and sew” reductive ascending aortoplasty (RAA): (**A**) Intraoperative view of a dilated ascending aorta. (**B**) Longitudinal S-shaped aortotomy along the greater curvature. (**C**) Completed RAA after wedge resection and double-layer continuous polyfilament suture closure (Blalock technique). Final appearance after aortoplasty.

For RAA, a wedge resection of the ascending aorta was performed through a longitudinal aortotomy starting at the greater curvature near the aortic clamp and extending toward the non-coronary sinus. A longitudinal S-shaped aortotomy was made along the greater curvature. The resulting elliptical segment of the anterior aortic wall was excised, and the aortotomy was closed linearly, thereby reducing the aortic diameter. The aorta was reconstructed using a double-layer continuous Ti-Cron 4-0 polyfilament suture (Medtronic Ltd., Watford, UK) (Blalock technique) (Figure 2). In selected cases, the suture line was reinforced with a Teflon felt band accompanied by fibrin glue along the suture line. The decision to reinforce the suture line with a Teflon felt band was made intraoperatively based on the perceived quality of the aortic tissue. In cases where the aortic wall appeared thin or fragile—most commonly in older patients or those with extensive medial degeneration—reinforcement was used to reduce the risk of suture line bleeding or pseudoaneurysm formation. Fibrin glue (Vivostat A/S, Alleroed, Denmark) was applied along the suture line in selected cases to achieve hemostasis and potentially reduce the risk of late redilatation by promoting tissue adherence.

### 2.5. Outcomes and Definitions

The primary endpoints were 30-day all-cause mortality, major adverse cardiac and cerebrovascular events (MACCEs; composite of death, myocardial infarction, stroke, or repeat revascularization), and 10-year overall survival.

Secondary outcomes included the incidence of aortic redilatation and the need for repeat surgery. Redilatation was defined as an ascending aortic diameter ≥ 50 mm during follow-up, representing a clinically relevant threshold for surveillance and potential reintervention.

LVEF < 30% was used as a threshold for severe left ventricular dysfunction, consistent with standard clinical definitions (ESC Heart Failure Guidelines, 2021, 2023 Focused Update) [16].

### 2.6. Imaging, Measurement, and Follow-Up Protocol

**Figure 3:**
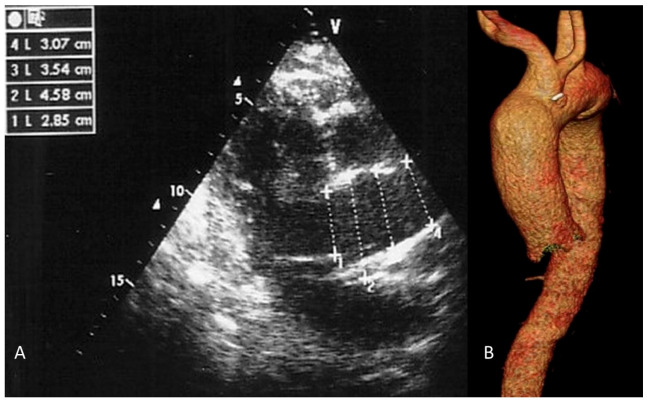
Postoperative imaging assessment at 12 months: (**A**) Transthoracic echocardiography (parasternal long-axis view) showing a reduced ascending aortic diameter (3.4 cm). (**B**) CT angiography (multiplanar reconstruction) confirming the stable aortic diameter after reductive aortoplasty.

Clinical follow-up included scheduled visits at 1, 6, and 12 months after surgery and annually thereafter. Surveillance was primarily performed using transthoracic echocardiography (TTE). Ascending aortic diameters were measured inner-edge to inner-edge in the parasternal long-axis view at the level of the pulmonary artery bifurcation during systole (Figure 3A). In cases of suboptimal echocardiographic windows, discrepancy between clinical and imaging findings, or TTE suggestion of a diameter ≥ 45 mm, computed tomography (CT) angiography was performed (Figure 3B). A total of 18 patients underwent 24 CT scans during follow-up.

All TTE measurements were performed by experienced echocardiographers using a standardized protocol. Measurement of the ascending aortic diameter was performed by an operator who was blinded to the clinical data.

Complete follow-up was defined as the availability of either clinical status (via in-person visit or structured telephone interview) or imaging data (TTE or CT) at the 10-year time point or until death. Patients followed by telephone interview underwent routine TTE at their local institution, with reports obtained for review. Complete follow-up was achieved for all 64 patients.

### 2.7. Statistical Analysis

Continuous variables are presented as mean ± standard deviation or median with interquartile range and compared using Student’s *t*-test or Mann–Whitney *U* test, as appropriate. Categorical variables were compared using Fisher’s exact test.

Early binary outcomes were analyzed using logistic regression with Firth penalization when event counts were sparse. Time-to-event outcomes (redilatation, reintervention, and survival) were analyzed using Cox regression with proportional hazard assumptions verified. Survival was estimated by Kaplan–Meier analysis with log-rank testing for between-group comparisons. Treatment effects are expressed as adjusted odds ratios or hazard ratios with 95% confidence intervals.

Multivariable models included age (continuous), sex, baseline aortic diameter, and relevant comorbidities. Variables included in the multivariable Cox regression were selected a priori based on clinical relevance (age, sex, and baseline aortic diameter) and univariable association with the outcome (*p* < 0.10). Sensitivity analyses were performed for age ≥ 70 years and with the exclusion of syndromic/connective tissue disease.

Post hoc power calculation for the primary outcome (aortic redilatation ≥ 50 mm) was performed using a two-sample proportion test. With a total sample size of 64 (19 BAV, 45 TAV) and an observed redilatation rate of 31.6% in BAV vs. 8.9% in TAV, the study had 80% power to detect a 20% difference in proportions at α = 0.05.

There were no missing data for the primary endpoints (mortality, MACCE, redilatation). For secondary outcomes, completeness was 100% for all variables included in the analysis.

Statistical analyses were conducted using SPSS version 26 (IBM Corp., Armonk, NY, USA) and R version 4.0 (R Foundation for Statistical Computing, Vienna, Austria; https://www.R-project.org/) . Specific outcomes, including causes of late mortality and types of aortic reintervention, are reported descriptively.

### 2.8. Ethical Considerations

The study protocol was developed in accordance with the Declaration of Helsinki. Written informed consent was obtained from all participants for both the surgical procedures and subsequent data collection. The independent institutional ethics committee approved the study protocol (reference number: 11.06.2005), including provisions for patient privacy and data security.

## 3. Results

### 3.1. Early Postoperative Outcomes

Considering the 64 patients (45 TAV, 19 BAV), the mean age was 64.5 ± 10.1 years (12 ≥ 70). Baseline characteristics were comparable (Table 1).

Preoperative aortic diameter was 5.5 ± 1.1 cm (TAV) vs. 5.4 ± 1.2 cm (BAV). Most of those with BAV had stenosis; patients with TAV had mixed lesions. Length of stay, cross-clamp times, and early complications were similar (Table 2).

All patients underwent valve replacement with concomitant unwrapped RAA. One early death occurred (TAV group). The mean postoperative aortic diameter was 3.4 ± 0.3 cm (Figure 3). Reexploration for bleeding occurred in 9.37% of the patients (6/64). The incidence was higher in the BAV group (15.79% [3/19]) compared to the TAV group (6.66% [3/45]), but this difference did not reach statistical significance (*p* = 0.109). Histological examination of the excised aortic wall was available for 58 patients (90.6%). Degenerative medial changes—including elastic fiber fragmentation, smooth muscle cell loss, and accumulation of ground substance (mucoid extracellular matrix deposition)—were observed in the majority of the patients (49/58, 84.5%). These changes were more prevalent in BAV patients (17/19, 89.5%) compared to TAV patients (32/45, 71.1%), consistent with the known aortopathy associated with bicuspid valve disease. Moderate-to-severe medial degeneration was observed in 12 patients (20.7%), all of whom had preoperative aortic diameters ≥ 55 mm.

### 3.2. Outcomes by Valve Phenotype

Of the 64 patients, 19 (29.7%) had BAV, and 45 (70.3%) had TAV. The baseline aortic diameter was similar between the groups (5.5 ± 1.1 cm BAV vs. 5.4 ± 1.2 cm TAV; *p* = 0.756). Cross-clamp and CPB times were similar (*p* = 0.153). There was no 30-day mortality in either cohort; major early complications did not differ.

During a mean follow-up of 9.3 ± 1.2 years, redilatation (≥50 mm) occurred in 6 BAV patients (31.6%) compared to 4 TAV patients (8.9%), representing a trend toward higher risk in BAV patients (RR = 3.55, 95% CI: 1.08–11.7; OR = 4.71, 95% CI: 1.21–18.4; *p* = 0.053). Aortic reintervention was required in 4 BAV patients (21.1%) and 3 TAV patients (6.7%) (RR = 3.15, 95% CI: 0.75–13.2; OR = 3.71, 95% CI: 0.77–17.9; *p* = 0.10) (Table 3). In multivariable analysis adjusted for age, sex, and baseline aortic diameter, BAV phenotype remained independently associated with redilatation (adjusted HR = 4.1, 95% CI: 1.1–15.3; *p* = 0.035). Sensitivity analysis excluding patients ≥ 70 years yielded consistent results.

### 3.3. Long-Term Clinical Outcomes

**Figure 4:**
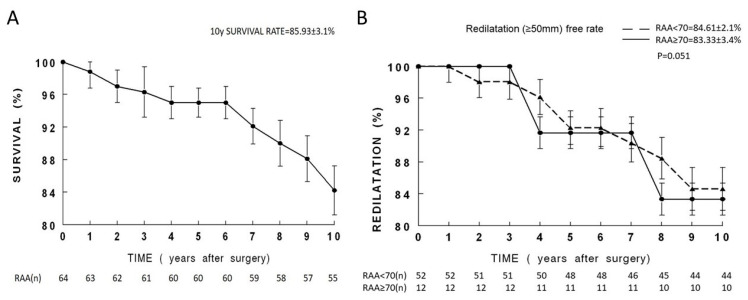
Long-term outcomes after reductive aortoplasty: (**A**) Ten-year overall survival for the entire cohort (N = 64). Mean follow-up: 9.3 ± 1.2 years, 100% complete. Number at risk below. (**B**) Freedom from redilatation (≥50 mm) by age: ≥70 years (n = 12) vs. <70 years (n = 52). Trend toward superior stability in the older cohort (log-rank *p* = 0.051). Number at risk below.

Complete follow-up was achieved for all 64 patients at a mean duration of 9.3 ± 1.2 years. Both groups demonstrated significant clinical improvement from baseline. At 10 years, survival was favorable in both groups, with no significant difference between TAV and BAV (Figure 4A). During long-term follow-up, redilatation of the ascending aorta (≥50 mm) occurred in a minority of patients and was more frequent among those with bicuspid aortic valve morphology. Age subgroup analysis (<70 vs. ≥70) showed similar redilatation rates between the groups (15.4% vs. 16.7%; RR = 0.92, 95% CI: 0.21–4.05; *p* = 1.00). However, all reinterventions occurred in patients < 70 years (7/52, 13.5%), with none in patients ≥ 70 years (Table 4, Figure 4B). No significant differences in MACCEs were observed.

Late mortality causes were similarly distributed, predominantly from aortic redilatation and the need for reintervention, cardiac failure, and cerebrovascular events. Specific causes of late mortality (n = 10) were cardiac failure (n = 4), cerebrovascular events (n = 3), malignancy (n = 2), and respiratory failure (n = 1). No late deaths were attributable to aortic dissection.

Aortic reinterventions (n = 7) were performed for redilatation ≥ 50 mm at a mean of 8.2 ± 2.1 years after the initial procedure. The indications for reintervention were progressive aortic dilatation with symptoms (n = 3), rapid growth (>5 mm/year) on surveillance imaging (n = 2), and asymptomatic dilatation reaching 55 mm (n = 2). All reinterventions were performed electively, and no patient required emergent surgery for aortic dissection or rupture. The procedures consisted of composite valve–graft replacement (Bentall, n = 3; all in BAV patients) and isolated ascending aortic graft replacement (n = 4; 1 BAV, 3 TAV). There were no perioperative deaths or major complications associated with reintervention.

During follow-up, a total of 218 transthoracic echocardiograms were performed (mean 3.4 ± 1.2 studies per patient). CT angiography was utilized in 18 patients (28%) based on the protocol, resulting in 24 total CT scans. The mean annual growth rate of the ascending aorta post-RAA was 0.7 ± 0.2 mm/year. At the final follow-up, 8 patients (12.5%) had an aortic diameter between 45 and 49 mm.

## 4. Discussion

This 20-year analysis of RAA provides critical long-term data to refine its role in contemporary practice. The results demonstrate that RAA can achieve favorable long-term outcomes in carefully selected patients, supporting its use as a tissue-preserving strategy with clearly defined indications and contraindications. Specifically, RAA offers excellent durability for older patients (≥70 years) with TAV, while the high rates of redilatation and bleeding in BAV patients argue strongly against its routine use in this population. This risk-stratified framework directly addresses the historical uncertainty surrounding patient selection for RAA.

Current guidelines recommend ascending aortic replacement when specific diameter thresholds are reached, particularly in BAV disease or when additional risk factors for dissection are present [3,4,5]. While prosthetic replacement provides durable mechanical stability, it inevitably abolishes the elastic properties of the aortic wall and carries increased procedural complexity. RAA, first introduced more than half a century ago, offers a technically simpler alternative that preserves native tissue and has the potential to maintain the physiological properties of the aortic root and proximal ascending aorta [8]. Despite these advantages, the procedure has largely fallen out of favor because of concerns regarding late redilatation and uncertain durability [17,18,19,20].

### 4.1. Early Outcomes and Safety

One death (1.6%) compares favorably with the 2–6% mortality reported in a recent series of AVR combined with ascending aortic replacement [17,18,19]. Notably, the single mortality in our series occurred in a TAV patient who developed postoperative low cardiac output syndrome despite preserved preoperative left ventricular function. This mortality rate is particularly encouraging, given that our cohort included patients with significant comorbidities (Logistic EuroSCORE II 3.6 ± 2.8) and a subset of high-risk patients in whom RAA was chosen as a risk-adapted alternative. MACCEs were rare, with no strokes or myocardial infarctions recorded, supporting the safety profile of RAA in this population. Reexplorations were for bleeding, not suture failure, and no pseudoaneurysms occurred. The non-significant trend toward more bleeding in BAV (15.8% vs. 6.7%, *p* = 0.109) aligns with known medial fragility [19] and warrants further study.

### 4.2. Long-Term Durability

Late redilatation was uncommon, supporting RAA durability in selected patients. Despite these favorable overall results, redilatation occurred more frequently in patients with bicuspid aortic valve morphology. Although the mean postoperative aortic diameter was effectively reduced to 3.4 ± 0.3 cm, the incidence of redilatation was significantly higher in BAV patients compared with those with tricuspid valves (31.6% vs. 8.9%; *p* = 0.053). Slow growth (0.7 mm/year) and progression to 45–49 mm in 12.5% suggest gradual rather than acute failure. No dissection/rupture occurred, supporting echocardiography-based surveillance with selective CT (used in 28%). This finding underscores that valve phenotype represents an important determinant of long-term durability after RAA.

The durability observed in the overall cohort—and particularly among patients with tricuspid aortic valves—likely reflects careful patient selection, consistent with prior reports demonstrating acceptable long-term outcomes when RAA is applied selectively [8,9]. Patients with connective tissue disorders or extensive aortic wall degeneration were excluded in accordance with guideline recommendations. By restricting the indication to degenerative aneurysmal dilatation in the setting of aortic valve disease, particularly when valve replacement was required, the risk of rapid recurrent enlargement was minimized.

### 4.3. Age-Dependent Outcomes

RAA’s success is influenced by age, though the relationship is nuanced. Patients ≥ 70 years demonstrated outstanding durability with no reinterventions, while 80% of redilatation events and all reinterventions occurred in patients < 70. Though absolute redilatation rates were similar between age groups (15.4% vs. 16.7%), the clinical implications differ profoundly. For older patients, procedural simplicity, age-related aortic fibrosis, and limited life expectancy make RAA preferable—avoiding graft complexity without compromising safety.

The biological mechanisms underlying this age-dependent success are multifactorial. With advancing age, the aortic wall undergoes progressive elastin fragmentation, increased collagen deposition, and smooth muscle cell atrophy—changes that result in a stiffer but mechanically more stable vessel [7]. This age-related fibrosis may paradoxically reduce the propensity for progressive dilatation after reduction aortoplasty, as the tissue is less compliant and therefore less likely to stretch over time. Furthermore, the limited life expectancy of older patients means that the 10–15-year durability demonstrated in this study may be sufficient for their remaining lifespan. This concept of “biological durability”—where a procedure needs only to outlast the patient—is particularly relevant in geriatric surgical decision-making.

For younger patients, particularly those with BAV, the higher risk of late aortic events mandates caution.

### 4.4. Valve Phenotype and Aortopathy

The observed differences between BAV and TAV patients are consistent with extensive literature demonstrating that BAV aortopathy is an intrinsic, genetically mediated process rather than a simple hemodynamic consequence of valve dysfunction [3,14,21]. Histologically, BAV aortopathy is characterized by greater elastic fiber fragmentation, smooth muscle cell loss, and increased matrix metalloproteinase activity compared to degenerative aneurysms in TAV patients. This more aggressive biology likely explains the higher rates of redilatation observed in our BAV cohort despite an effective initial reduction in aortic diameter (31.6% vs. 8.9%; OR 4.71, *p* = 0.053). The fact that 21.1% of BAV patients ultimately required reintervention (vs. 6.7% in TAV), most requiring root replacement, suggests that RAA may be insufficient to address the underlying aortopathy in this population. RAA should be avoided in most BAV patients with substantial life expectancy. Larger series with dedicated BAV cohorts are required to precisely define the risk profile and identify which BAV patients might still be suitable candidates for RAA.

### 4.5. Physiological Considerations: The Windkessel Effect

An important aspect of this debate is the physiological role of the ascending aorta as an active component of the cardiovascular system. Recent consensus increasingly conceptualizes the ascending aorta as a biologically active organ with regulatory and buffering functions. This concept is supported by mechanistic studies demonstrating region-specific stress responses, microstructural remodeling, and oxidative signaling within the aortic wall, differing by valve phenotype [14]. Through the Windkessel effect, the aorta absorbs part of the stroke volume during systole and releases it during diastole, maintaining continuous flow and ensuring coronary perfusion. Dilatation and stiffening impair this effect, leading to widened pulse pressure, increased left ventricular afterload, and compromised myocardial perfusion [13].

Prosthetic graft replacement, while structurally effective, abolishes this property because woven polyester grafts are rigid and noncompliant. In contrast, RAA preserves native tissue and may retain some physiological elasticity, partially restoring the Windkessel effect. Although our study was not designed to measure hemodynamic indices directly, the long-term clinical stability observed may in part reflect the preservation of this physiological buffering function. This concept represents an important area for future research, potentially involving advanced imaging and compliance measurements.

### 4.6. Limitations

The principal limitations of this study are its retrospective, single-center design and the small sample size, especially within the BAV subgroup (n = 19). The single-arm, non-comparative design means that the outcomes are descriptive, and any comparisons to the standard of care (graft replacement) are indirect and based on historical literature. Propensity matching was not performed due to the small sample size, but the groups were comparable at baseline (Table 1). Furthermore, our follow-up relied primarily on transthoracic echocardiography, supplemented by CT angiography in selected patients. While consistent with current practice, transthoracic echocardiography is known to underestimate ascending aortic diameter compared with CT or MRI. However, consistent use of a standardized inner-edge measurement protocol across all patients minimized this limitation. Nonetheless, follow-up completeness was excellent, with data available for all patients up to 10 years.

## 5. Clinical Implications and Conclusions

Based on our findings, we propose the following patient selection framework for RAA. The procedure is strongly indicated for patients ≥ 70 years old with TAV and an aorta of 45–55 mm, in whom it offers excellent durability as a less invasive, tissue-preserving strategy. RAA is relatively contraindicated in patients < 70 years with TAV and should only be considered if life expectancy is limited or comorbidities drastically increase graft replacement risk. Most importantly, RAA should be avoided in patients with BAV aortopathy except in the most extreme high-risk scenarios, given the high rates of redilatation (31.6%) and reintervention (21.1%) observed in this population. Absolute contraindications remain connective tissue disorders or rapid preoperative growth (>5 mm/year).

This long-term, risk-stratified assessment moves the discussion beyond whether RAA is feasible to defining for whom it is appropriate: older TAV patients are ideal candidates, while BAV patients are better served by prosthetic graft replacement.

## Figures and Tables

**Table 1:** Clinical characteristics of the RAA population.

Parameter	BAV (n = 19)	TAV (n = 45)	*p*
**Gender**			
male	12 (63.15%)	32 (71.11%)	0.685
**Mean age**	65.5 ± 10.1	61.9 ± 10.8	0.881
<70	17 (89.47%)	35 (77.78%)	0.783
≥70	2 (10.52%)	10 (22.22%)	1.00
**Baseline Ao diameter**	55 ± 1.1	54 ± 1.2	0.756
**EF mean**	51.21 ± 13.4%	49.56 ± 7.2%	0.743
EF < 30%	3 (15.78%)	7 (15.55%)	1.00
EF > 30%	16 (84.21%)	38 (84.45)	1.00
**COPD**	2 (10.52%)	5 (11.11%)	1.00
**Diabetes**	2 (10.52%)	5 (11.11%)	1.00
**BMI**	27.4 ± 2.3	28.6 ± 3.1	0.824
**Valve lesions**			
Aortic insufficiency	2 (10.52%)	18 (40.0%)	0.834
Aortic stenosis	17 (89.47%)	27 (60.0%)	0.331
**Additional procedures**			
CABG	2 (10.52%)	10 (22.22%)	1.00
Mitral valve surgery	0	1 (2.22%)	1.00
**Logistic Euro SCORE II**	3.65 ± 2.8	3.5 ± 1.9	0.888

RAA = reductive ascending aortoplasty. COPD = chronic obstructive pulmonary disease; BMI = body mass index; CABG = coronary artery bypass grafting.

**Table 2:** Operative and early (30 days) outcomes in RAA patients.

Parameter	BAV (n = 19)	TAV (n = 45)	*p*
**Postoperative RAA diameter (cm)**	3.42 ± 0.8	3.49 ± 0.5	0.823
**Cross clamp time (min)**	77.63 ± 10.5	67.06 ± 11.2	0.153
**CPB time (min)**	65.54 ± 21.09	79.38 ± 11.8	0.679
**Average length of stay** (days)	7.01 ± 7.6	7.31 ± 0.5	0.914
**Reexploration for bleeding**	3/19 (15.8%)	3/45 (6.66%)	0.109
**MACCE**			
Death, stroke, or MI	0	1(2.22%)	1.00
Stroke	0	0	1.00
MI	0	0	1.00
			1.00
**Total mortality**	0	1(2.22%)	1.00
Cardiac	0	0	1.00
Non cardiac	0	1(2.22%)	1.00

RAA = reductive ascending aortoplasty; MACCE = major adverse cardiovascular and cerebrovascular events; CPB = cardiopulmonary bypass.

**Table 3:** Long-term outcomes (10-year follow-up) by valve phenotype.

Outcome	BAV (n = 19)	TAV (n = 45)	*p*-Value
**Redilatation ≥ 50 mm**			
	6/19 (31.6%)	4/45 (8.9%)	0.053
**Aortic reintervention**			
	4/19 (21.1%)	3/45 (6.7%)	0.182
**Overall survival**	**16/19 (84.21%)**	**39/45 (86.7%)**	1.00

BAV = bicuspid aortic valve; TAV = tricuspid aortic valve. Values are n/N (%) within each group. *p*-values by Fisher’s exact test; exploratory due to small strata.

**Table 4:** Long-term outcomes (10-year follow-up) by age.

Outcome	<70 (n = 52)	≥70 (n = 12)	*p*-Value
**Redilatation ≥ 50 mm**			
	8/52 (15.38%)	2/12 (16.66%)	1.00
**Aortic reintervention**			
	7/52 (13.46%)	0/12 (0.0%)	0.331
**Overall survival**	**44/52 (84.62%)**	**10/12 (83.33%)**	1.00

Values are n/N (%) within each group. *p*-values by Fisher’s exact test; exploratory due to small strata.

## Data Availability

The raw data supporting the conclusions of this article will be made available by the authors upon request.

## References

[1] Liu S., Shi Y., Liu R., Tong M., Luo X., Xu J. (2017). Early Prognosis of Reduction Ascending Aortoplasty in Patients with Aortic Valve Disease: A Single Center’s Experience. Ann. Thorac. Surg..

[2] Polvani G., Barili F., Dainese L., Topkara V.K., Cheema F.H., Penza E., Ferrarese S., Parolari A., Alamanni F., Biglioli P. (2006). Reduction ascending aortoplasty: Midterm follow-up and predictors of redilatation. Ann. Thorac. Surg..

[3] Hiratzka L.F., Creaguer M.A., Isselbacher E.M., Svensson L.G., Nishimura R.A., Bonow R.O., Guyton R.A., Sundt T.M. (2016). Surgery for aortic dilatation in patients with bicuspid aortic valves: A statement of clarification from the American College of Cardiology/American Heart Association Task Force on Clinical Practice Guidelines. J. Am. Coll. Cardiol..

[4] Czerny M., Grabenwöger M., Berger T., Aboyans V., Della Corte A., Chen E.P., Desai N.D., Dumfarth J., Elefteriades J.A., Etz C.D. (2024). EACTS/STS Guidelines for diagnosing and treating acute and chronic syndromes of the aortic organ. Eur. J. Cardiothorac. Surg..

[5] Mazzolai L., Teixido-Tura G., Lanzi S., Boc V., Bossone E., Brodmann M., Bura-Rivière A., De Backer J., Deglise S., Della Corte A. (2024). 2024 ESC Guidelines for the management of peripheral arterial and aortic diseases. Eur. Heart J..

[6] Clouse W.D., Hallett J.W., Schaff H.V., Gayari M.M., Ilstrup D.M., Melton L.J. (1998). Improved prognosis of thoracic aortic aneurysms: A population-based study. J. Am. Med. Assoc..

[7] Pisano C., Balistreri C.R., Ricasoli A., Ruvolo G. (2017). Cardiovascular Disease in Ageing: An Overview on Thoracic Aortic Aneurysm as an Emerging Inflammatory Disease. Mediat. Inflamm..

[8] Robicsek F. (1982). A new method to treat fusiform aneurysms of the ascending aorta associated with aortic valve disease: An alternative to radical resection. Ann. Thorac. Surg..

[9] Kiessling A.H., Odwody E., Miskovic A., Stock U.A., Zierer A., Moritz A. (2014). Midterm follow up in patients with reduction ascending aortoplasty. J. Cardiothorac. Surg..

[10] Carrel T., von Segesser L., Jenni R., Gallino A., Egloff L., Bauer E., Laske A., Turina M. (1991). Dealing with dilated ascending aorta during aortic valve replacement: Advantages of conservative surgical approach. Eur. J. Cardiothorac. Surg..

[11] Robicsek F., Cook J.W., Reames M.K., Skipper E.R. (2004). Size reduction ascending aortoplasty: Is it dead or alive?. J. Thorac. Cardiovasc. Surg..

[12] Kaneko T., Shekar P., Ivkovic V., Longford N.T., Huang C.-C., Sigurdsson M.I., Neely R.C., Yammine M., Ejiofor J.I., Vieira V.M. (2018). Should the dilated ascending aorta be repaired at the time of bicuspid aortic valve replacement?. Eur. J. Cardiothorac. Surg..

[13] Humphrey J.D., Milewicz D.M., Tellides G., Schwartz M.A. (2014). Cell biology. Dysfunctional mechanosensing in aneurysms. Science.

[14] Huckaby L.V., Fortunato R.N., Emerel L.V., Phillippi J.A., Billaud M., Vorp D.A., Maiti S., Gleason T.G. (2025). Wall Tensile Stress Maps of Human Aneurysmal Aorta Demonstrate a High Biaxiality Ratio Corresponds with Wall Tissue Microstructure and Local Oxidative Stress Response Distinctly for Bicuspid and Tricuspid Aortic Valve Patients. Ann. Biomed. Eng..

[15] Tremblay D., Leask R.L. (2011). Remodelling and pathology development associated with aneurysmal ascending aortic tissues. Can. J. Chem. Eng..

[16] McDonagh T.A., Metra M., Adamo M., Gardner R.S., Baumbach A., Böhm M., Burri H., Butler J., Čelutkienė J., Chioncel O. (2024). 2023 Focused Update of the 2021 ESC Guidelines for the diagnosis and treatment of acute and chronic heart failure: Developed by the task force for the diagnosis and treatment of acute and chronic heart failure of the European Society of Cardiology (ESC) with the special contribution of the Heart Failure Association (HFA) of the ESC. Eur. J. Heart Fail..

[17] Davies R.R., Goldstein L.J., Coady M.A., Tittle S.L., Rizzo J.A., Kopf G.S., Elefteriades J.A. (2002). Yearly rupture or dissection rates for thoracic aortic aneurysms: Simple prediction based on size. Ann. Thorac. Surg..

[18] Zhang H., Lu F., Qu D., Han L., Xu J., Ji G., Xu Z. (2011). Treatment of fusiform ascending aortic aneurysms: A comparative study with 2 options. J. Thorac. Cardiovasc. Surg..

[19] Dumani S., Pellumbi D., Likaj E., Dibra L., Rruci E., Kuci S., Llazo S., Mehmeti A., Refatllari A., Veshti A. (2025). Isolated Aortic Valve Replacement Versus Concomitant Replacement of the Ascending Aorta and Aortic Valve: A Statistical Analysis and Literature Review. Cureus.

[20] Matsuyama K., Usui A., Akita T., Yoshikawa M., Murayama M., Yano T., Takenaka H., Katou W., Toyama M., Okada M. (2005). Natural history of a dilated ascending aorta after aortic valve replacement. Circ. J..

[21] Girdauskas E., Disha K., Borger M.A., Kuntze T. (2014). Long-term prognosis of ascending aortic aneurysm after aortic valve replacement for bicuspid versus tricuspid aortic valve stenosis. J. Thorac. Cardiovasc. Surg..

